# Gene Correlation Network Analysis to Identify Biomarkers of Peri-Implantitis

**DOI:** 10.3390/medicina58081124

**Published:** 2022-08-19

**Authors:** Binghuan Sun, Wei Zhang, Xin Song, Xin Wu

**Affiliations:** Department of Stomatology, Nanjing First Hospital, Nanjing Medical University, 68 Changle Road, Nanjing 210006, China

**Keywords:** biomarkers, peri-implantitis, weighted gene co-expression network analysis (WGCNA)

## Abstract

*Background and Objectives*: The histopathological and clinical conditions for transforming peri-implant mucositis into peri-implantitis (PI) are not fully clarified. We aim to uncover molecular mechanisms and new potential biomarkers of PI. *Materials and Methods*: Raw GSE33774 and GSE57631 datasets were obtained from the Gene Expression Omnibus (GEO) database. The linear models for microarray data (LIMMA) package in R software completes differentially expressed genes (DEGs). We conducted a weighted gene co-expression network analysis (WGCNA) on the top 25% of altered genes and identified the key modules associated with the clinical features of PI. Gene Ontology (GO) enrichment and Kyoto Encyclopedia of Genes and Genomes (KEGG) pathway analyses were performed using the R software. We constructed a protein–protein interaction (PPI) network through the STRING database. After that we used Cytohubba plug-ins of Cytoscape to screen out the potential hub genes, which were subsequently verified via receiver operating characteristic (ROC) curves in another dataset, GSE178351, and revalidation of genes through the DisGeNET database. *Results*: We discovered 632 DEGs (570 upregulated genes and 62 downregulated genes). A total of eight modules were screened by WGCNA, among which the turquoise module was most correlated with PI. The Cytohubba plug-ins were used for filtering hub genes, which are highly linked with PI development, from the candidate genes in the protein–protein interaction (PPI) network. *Conclusions*: We found five key genes from PI using WGCNA. Among them, ICAM1, CXCL1, and JUN are worthy of further study of new target genes, providing the theoretical basis for further exploration of the occurrence and development mechanism of PI.

## 1. Introduction

Peri-implantitis (PI) is an inflammation of peri-implant mucosa with the plaque as the initiating factor and progressive bone loss around the implant [1]. With the globally increasing number of missing teeth restored with implants, PI is becoming a notable problem in implant dentistry [2]. According to the literature examined, the incidence of PI 5 to 10 years after implant placement seems to vary between 10 and 20 percent of implants and patients [3]. It has been revealed that PI is associated with microorganisms, individual genetic susceptibility, traumatic occlusal forces, diffusion of titanium particles in the adjacent tissues, and the biological reactions they provoke with excessive bonding adhesive after implant placement [4]. However, the histopathological and clinical conditions for transforming implant mucositis into PI are not fully clarified [5], and current treatments have limited success in improving the inflammatory environment of PI [6,7].

Numerous studies have been undertaken on the pathophysiology of PI. Some researchers have found that the pathogenesis of PI is caused by tissue damage mediated by the host’s immune response [8]. Pathogens trigger early innate immune responses in immune cells such as macrophages, neutrophils, dendritic cells, and Langerhans cells [9]. Stimulation of the innate immune system also induces an adaptive immunological response, as shown by an increase in B and T lymphocytes in the peri-implant tissues [10]. This innate and adaptive immunity is accompanied by an abundance of cytokines and chemokines produced in the peri-implant tissues, which, in a fine balance, help to maintain a healthy state [11]. However, some of them—such as IL-1β, tumor necrosis factor-α, IL-6, and IL-17—are thought to be potent proinflammatory molecules that may lead to tissue destruction if not properly controlled. These signaling molecules stimulate enzymes’ activation and local inflammation’s constant transcription cycle. Disruption of balance eventually leads to PI, resulting in bone loss and implant failure [12]. Significantly, there are many inconsistencies in the research results of PI-associated biomarkers. Some people succeeded in finding possible associations, while others did not. In addition, the pathogenesis of PI has not been fully revealed yet. Therefore, more advanced methods and more research are needed to validate the markers of PI and further reveal the pathogenesis of the disease.

For example, WGCNA [13] may be generated to identify gene modules, clinical characteristics (such as the severity of the illness), and the core genes included inside each module. It is also possible to build a non-coding RNA co-expression network as well as the miRNA and lncRNA networks. At present, there are few kinds of studies that research the pathogenesis of PI in this way. Yang Li et al. [14] believed that GSK3B and Mir-1297 might have a major impact on the immune microenvironment and pathogenesis of PI using WGCNA. To obtain a deeper understanding of co-expression in PI, we performed the WGCNA in this study to uncover the processes behind novel biomarkers of PI.

## 2. Materials and Methods

### 2.1. Data Collecting

According to the information included in the Gene Expression Omnibus (GEO) database [15] (https://www.ncbi.nlm.nih.gov/geo/ accessed on 15 February 2022), we downloaded the PI-related GSE33774 and GSE57631 genome-wide expression datasets. The GSE33774 dataset included 8 healthy samples and 7 PI samples. GSE33774 platform is GPL6244 [HuGene-1_0-st] Affymetrix Human Gene 1.0 ST Array [transcript (gene) version]. The GSE57631 dataset comprised 2 healthy samples and 6 PI samples, and the platform is GPL15034 [HuGene-1_0-st] Affymetrix Human Gene 1.0 ST Array [HuGene10stv1_Hs_ENTREZ, Brainarray v14].

### 2.2. Identification of DEGs

We applied the LIMMA package [16] and the robust multi-array average (RMA) technique in the R software for normalizing the data and analyzing differentially expressed genes (DEGs) based on the dataset GSE57631 raw data files. Genes satisfying log2 Fold Change absolute value > 1 and *p* value < 0.05 were screened as differentially expressed genes (DEGs).

### 2.3. WGCNA Generates a Network of Co-Expression

Using WGCNA analysis in the “R” tool, gene co-expression networks were created, and significant modules associated with the clinical features of PI were discovered. We established the soft threshold β using the range from 1 to 20. By modifying the weighted adjacency matrix, a topological overlap measure (TOM) matrix was produced. Comparing the TOM dissimilarity degree of genes with identical expression patterns led to the formation of distinct modules, and a cluster tree was constructed using the linkage hierarchical clustering strategy. For modules related to clinical attributes, we set the minimum gene number of modules to be greater than 200, and modules with dissimilarity degrees lower than 0.25 were combined [17]. The module most associated with PI was screened, and the module membership (MM) and gene correlation (GS) were calculated. Common genes (CGs) refer to the intersection of genes in significant gene modules and DEGs.

### 2.4. Functional Enrichment of Candidate Genes

For CGs, GO annotation in the R software package org.hs.eg. db (Version 3.1.0, Marc Carlson, Washington, DC, USA) was used to perform functional enrichment analysis. It consists of biological processes, cell composition, and molecular function. We concurrently utilized the KEGG rest application programming interface (API) for the most recent KEGG pathway gene annotation as background, mapping genes to the background in the collection. For enrichment analysis, the R software package clusterProfiler (Version 3.14.3, Yu G et al., Guangzhou, China) was used. The KEGG reference website is as follows (https://www.kegg.jp/kegg/rest/keggapi.html accessed on 27 February 2022).

### 2.5. PPI Network Creation and Displaying Hub Genes

We built the PPI interaction network of genes using the STRING resource. (https://cn.string-db.org/ accessed on 10 March 2022). The interaction score ≥0.4 was defined as the minimum value. Network visualization and hub genes identification were performed via making use of the program Cytoscape (version 3.8.0, San Diego, CA, USA) together with the GeneMANIA and Cytohubba plug-in [18].

### 2.6. ROC Validation of the GSE178351 Dataset and Validation from the DisGeNET Database

We used the pROC package and GGploT2 package in the R tool (version 3.6.3, Ross Ihaka and Robert Gentleman, Auckland, New Zealand) for analyzing and graphically confirming the hub genes from the training dataset GSE178351. Besides, DisGeNET (http://www.disgenet.org accessed on 30 May 2022) is a database developed to help researchers overcome disease barriers. It collects many related human diseases [Mendelian, complex and Environmental diseases (Piñero et al. Barcelona, Spain)], related variants and genes. Through the information provided by the database, we comprehensively analyzed the accuracy and scientificity of the screening target genes of PI.

## 3. Results

### 3.1. Identification of DEGs

**Figure 1:**
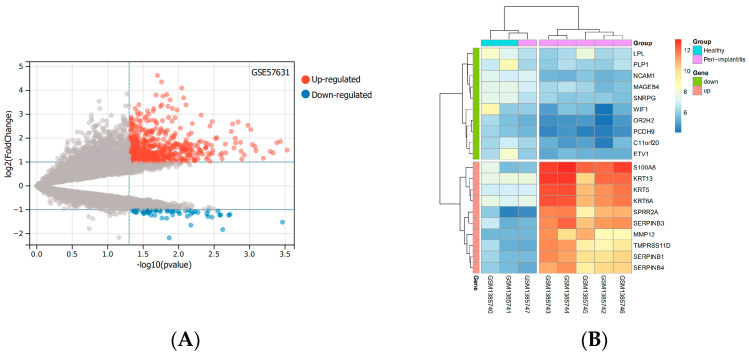
DEGs based on the GSE57631. (**A**) The volcano diagram of up and down DEGs. Grey dots represent genes that are not differentially expressed or statistically significant. (**B**) The heatmap of the top 10 significantly up− or down-regulated genes with the largest differential multiples. (Abbreviations: DEGs, differentially expressed genes; GSE, GEO Series; GEO, Gene Expression Omnibus).

Based on the mRNA expression profile data in the GSE57631 dataset. There were 632 DEGs between PI and healthy controls, between them, there were 570 identified genes with an increase in expression and 62 identified genes with a decrease in expression. The top 10 up-and down-regulated genes with the most pronounced differences were shown by the heatmap. The levels of expression of these DEGs varied considerably between the two groups (Figure 1).

### 3.2. Build a Scale-Free Network through WGCNA

**Figure 2:**
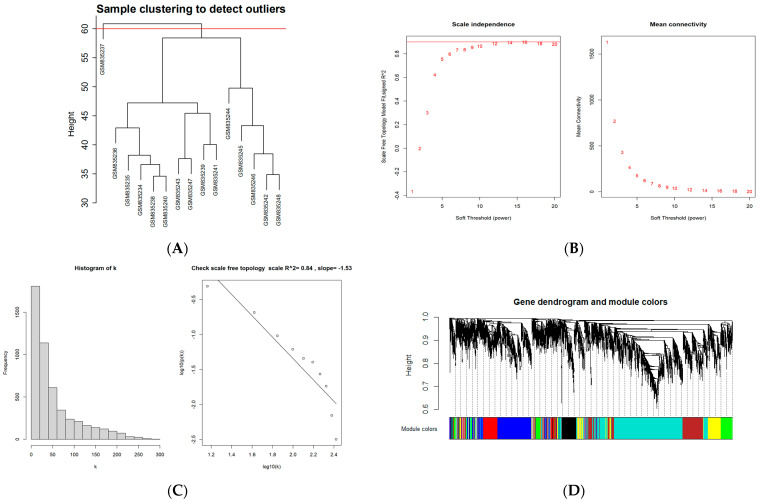

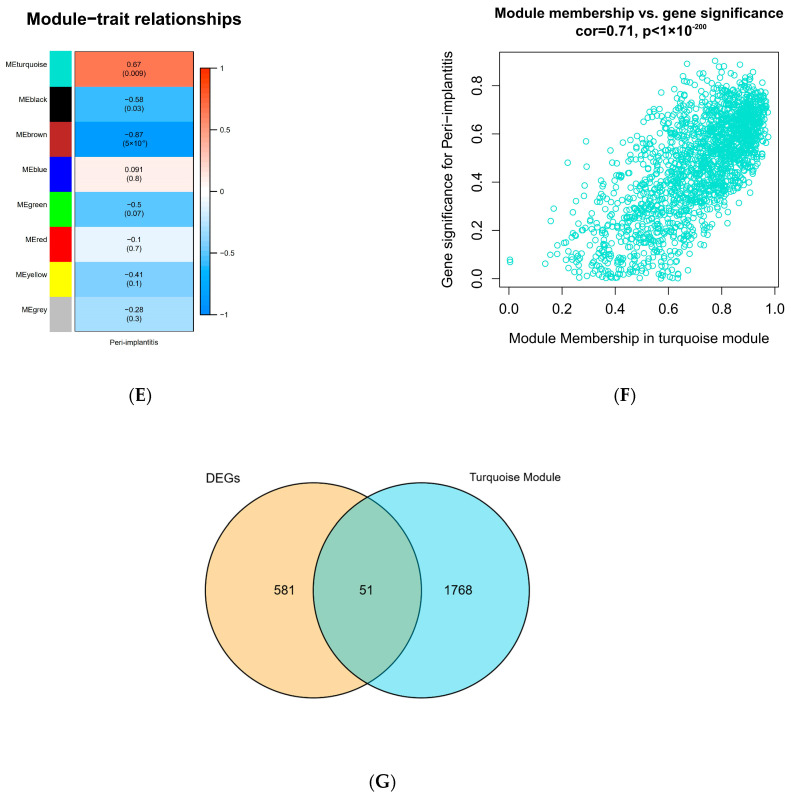
WGCNA diagram. (**A**) Sample clustering. (GSM835237 was removed from the queue) (**B**,**C**) Selection of soft-threshold β. (**D**) Clustering dendrogram. (**E**) Heatmap of the correlation between modules and clinical characteristics. (**F**) PI gene significance vs. membership in the turquoise module is shown in a scatter plot. (**G**) Venn diagram for screening CGs. (Abbreviations: CGs, common genes).

To construct a scale-free weighted co-expression network, we selected the top 25% of standard deviation genes from the GSE33774 dataset for WGCNA. After clustering the samples, an obvious outlier (GSM835237) was removed from the queue. In the range of values from 1 to 20, β = 9 was determined as the optimal soft threshold by the function “SFT$PowerEstimate” to construct a network without scale with a fitting index of more than 0.85. Additionally, the weighted adjacency matrix is converted into a topological overlap measure (TOM) matrix in order to illustrate gene connectivity in the network. We constructed a hierarchical clustering tree between genes based on TOM, and the modules with high MEs similarity were combined by the dynamic shearing method, and eight co-expression modules were derived. The maximum modulus is 1819 genes (turquoise), and the minimum modulus is 321 genes (black). The genes in the grey module represent those not included in any of the modules. We found that the modular characteristic genes (MEs) in the turquoise module (R = 0.67; P = 0.009) were closely associated with the disease compared with other modules. In addition, the correlations between gene module members (MM, association between a particular gene and module-specific genes) and gene significance (GS, connection between clinical factors and a specific gene) were calculated in the turquoise module. A significant positive association existed between MM and GS (COR = 0.71, P = 1 × 10^−200^). The intersection of the DEGs with the genes in the turquoise module is the common genes (CGs) (Figure 2).

### 3.3. Function and Pathway Enrichment Analysis of Common Genes

**Figure 3:**
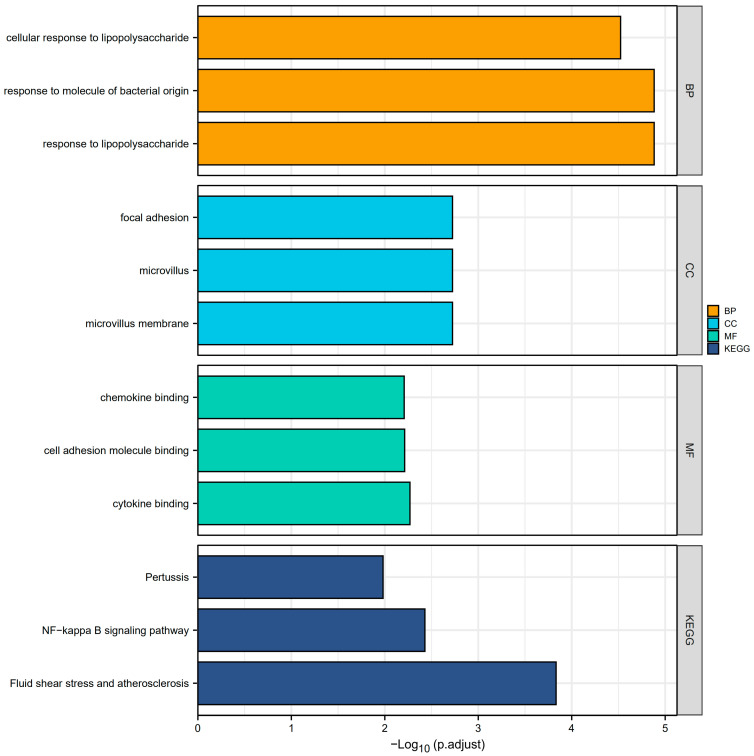
GO enrichment and KEGG analyses. The plot for the top 3 terms of GO enrichment and KEGG analyses. (Abbreviations: GO, Gene Ontology; KEGG, Kyoto Encyclopedia of Genes and Genomes; BP, biological process; CC, cellular component; MF, molecular function).

Overall, A total of 51 CGs associated with PI were obtained after the intersection of the DEGs and the genes in the turquoise module. These genes were analyzed for functional enrichment. GO enrichment analysis includes biological process, cell composition, and molecular function. As shown in Table 1, the cellular reaction to lipopolysaccharide, response to molecules of bacterial origin, and response to lipopolysaccharide are involved primarily in the biological processes (BP). Among them, the cellular reaction to lipopolysaccharides is a branch of response to lipopolysaccharides. This can also affect the cellular response to molecules of bacterial origin, the cellular response to oxygen-containing compounds, and the cellular response to lipids, based on the explanation from the GO annotations. The cell composition (CC) primarily consists of focal adhesion, microvillus, and microvillus membrane. Molecular functions (MF) include chemokine binding, cell adhesion molecule binding, cytokine binding, etc. In addition, the KEGG pathway enrichment analysis revealed that molecules are most abundant in the NF-kappa B signaling network, Fluid shear stress, atherosclerosis, and Pertussis pathways (Figure 3).

### 3.4. Discovery and Characterization of Hub Genes in the Protein–Protein Interaction Network

**Figure 4:**
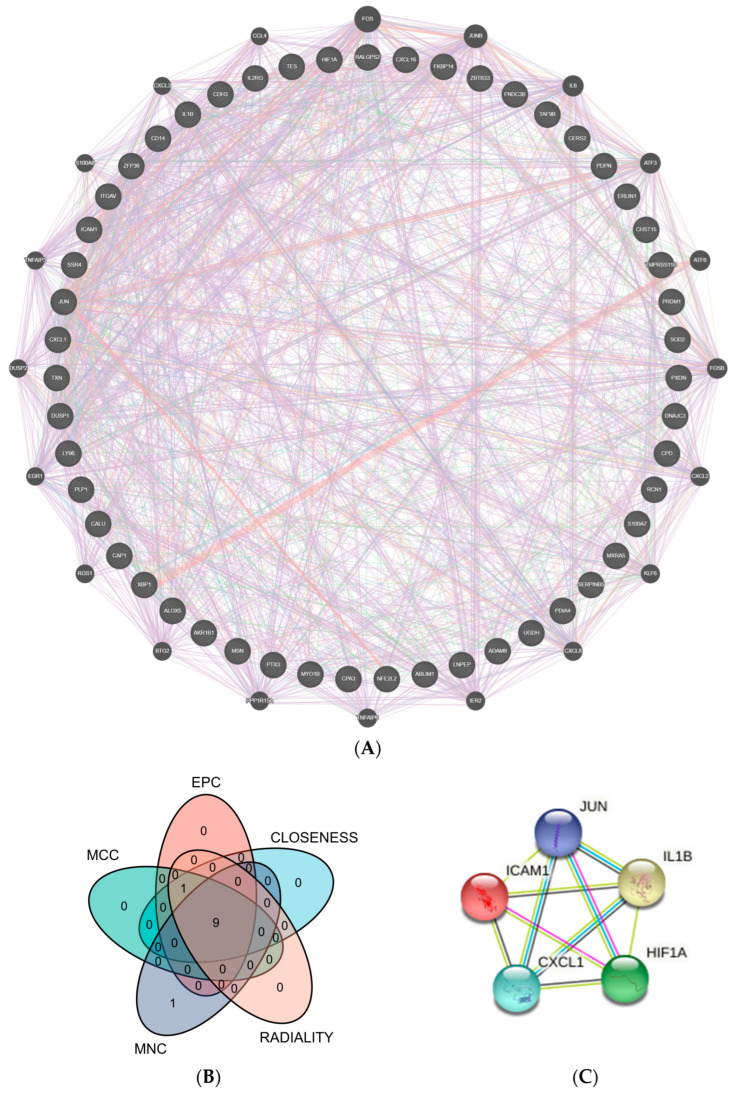
PPI network. (**A**) 51 crucial common genes in PPI network. (**B**) Venn diagram displaying the intersection of five algorithms in Cytohubba with the top 10 genes (EPC, MNC, MCC, Closeness, and Radiality). (**C**) Top 5 hub genes were predicted by the STRING database. (Abbreviations: PPI, protein–protein interaction; MCC, Maximal Clique Centrality; EPC, Edge Percolated Component; MNC, Maximum Neighborhood Component).

The CGs’ PPI interconnection network was constructed from the STRING database. The proteins independent of the network of interactions were removed, and those interacting were retained. We used Cytoscape and the GeneMANIA plug-in to visualize the interactions between genes. According to the five algorithms (EPC, MNC, MCC, Closeness, and Radiality) in the Cytohubba plug-in, the top 10 genes were intersected. The top 5 genes were selected as hub genes from inserted genes by ranking. They are IL1B, ICAM1, JUN, HIF-1α, and CXCL1 (Figure 4).

### 3.5. Multiple Validations of Hub Genes

**Figure 5:**
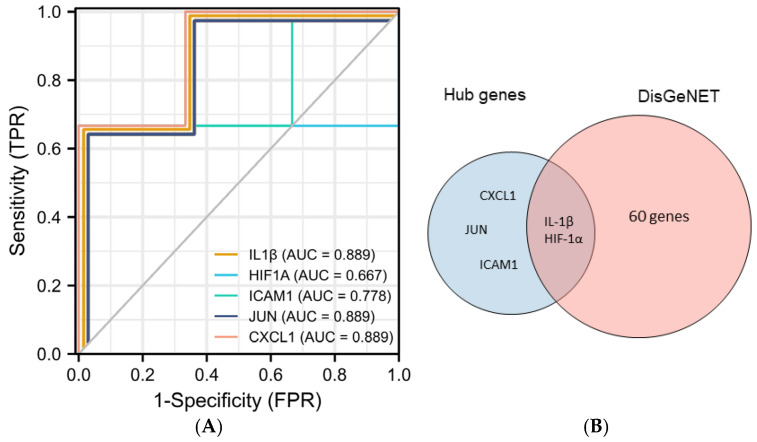
Validation of hub genes. (**A**) Predicted ROC curves of hub genes based on GSE178351. (**B**) Venn diagram of intersecting genes of top 5 genes and PI-related genes from the DisGeNET database.

Five hub genes (IL1B, ICAM1, JUN, HIF-1α, and CXCL1) were selected to further explore their correlation with clinical value. In predicting healthy and PI outcomes, IL-1β had certain precision (AUC = 0.889, CI = 0.581–1.000). HIF-1α had a moderate accurateness. (AUC = 0.667, CI = 0.013–1.000) The predictive ability of ICAM1 had some fidelity (AUC = 0.778, CI = 0.291–1.000). The predictive ability of JUN and CXCL1 had certain efficiency (AUC = 0.889, CI = 0.581–1.000). In addition, we found that 62 genes were closely related to the development of PI from the DisGeNET database. Among them, IL1B and HIF-1α are contained in the database, and the other three genes, ICAM1, CXCL1, and JUN, have not yet been included, which may suggest that they are potential biomarkers of PI (Figure 5).

## 4. Discussion

Dental implants have been used routinely for in treating edentulous and partly edentulous patients for over 25 years [19]. The annual number of implants implanted has risen enormously as treatment methods developed more predictable and successful [20]. However, the overall number of patients who suffered from PI rose as well.

The prognosis of PI mostly depends on the early detection and treatment of PI [2,21]. We choose bioinformatics to address this issue. Bioinformatics is the research of biological data using computer tools [22]. The method is based on bioinformatics interpretation and data processing to find out how the biological system of disease works. [23].

In this study, we used whole gene expression profile data to reveal the potential mechanism and markers of PI by WGCNA. First, based on clinical characteristics, eight identified modules were screened between the PI group and the healthy group. The turquoise module was closely correlated with PI. We identified 51 potential genes as 632 DEGs from another dataset (GSE57631) that intersected with this module. Among the GO categories, inflammatory and immunological responses were found to be the most common categories. KEGG pathway analysis indicates that the genes were primarily enriched on fluid shear stress, atherosclerosis pathway, and the NF-kappa B signaling pathway. It is noted that periodontitis and periodontal pathogens are associated with systemic diseases, such as cardiovascular disease. Porphyromonas gingivalis, for example, degrades gingivalprotease, vascular endothelial adhesion protein, and platelet endothelial cell adhesion molecule-1, leading to vascular endothelial damage and affecting the integrity of the endothelium. Thus, they caused endothelial dysfunction and possibly caused atherosclerotic cardiovascular disease [24]. Furthermore, our findings indicate that patients with pertussis disease may be more susceptible to PI. Fan et al. believe that immunizing patients with Bordetella pertussis vaccine could effectively prevent alveolar bone loss in PI, as demonstrated through experimental studies [25]. Since vaccination successfully avoids pertussis infection, little study has been conducted on this subject.

Then, we used the program Cytoscape (version 3.8.0, San Diego, CA, USA) together with the Cytohubba plug-in screening process for identifying five hub genes, namely IL-1β, ICAM1, JUN, HIF-1α, and CXCL1.

Based on the results, we found that IL-1β was the most significant biomarkers in PI-related biomarker. IL-1β is a pro-inflammatory cytokine in healthy peri-implant tissues at concentrations that reflect the true level of peri-implant tissue inflammation [26]. It plays a key role in bone resorption and extracellular matrix destruction by increasing the level of matrix metalloproteinase (MMP). In addition, it stimulates the proliferation of fibroblasts in inflammatory periodontal tissue and the hemagglutination of neutrophils to induce endothelial cells, regulating the degradation of connective tissue and repair activity [27]. It is the subject of varying scientific opinions whether IL-1β may be used as a biomarker for PI. We demonstrated that the increased IL-1β expression in the vicinity of PI causes local or systemic inflammation mainly through the NF-kB signaling pathway, which is a conventional pro-inflammatory signaling pathway whose presence is advantageous for immunological function, osteogenesis, and osteolysis [28].

HIF-1α is a key protein that regulates the response of multiple genes to hypoxia stimulation. The levels of HIF-1 were associated with site-specific clinical periodontal characteristics in PI patients [29]. The high level of HIF-1α immunostaining in PI suggests that hypoxia may be the pathogenesis of peri-implant diseases and bacterial infection [30].

ICAM1 is an adhesion receptor and cell surface glycoprotein that plays a crucial function in inflammatory tissues. Epithelial and immune cells strongly induce ICAM-1 expression under inflammatory stimulation, which plays an essential role in efferocytosis processes. It eliminates necrotic and apoptotic cells and reprograms tissue-resident cells to a pre-lysis phenotype to achieve the goal of removing inflammation and healing wounds by recruiting inflammatory macrophages [31]. In the initial stage of PI active capillary loops and inflammatory infiltrates can be detected in the connective tissue of the junctional epithelium and the apex of the sulcular epithelium. The presence of ICAM1 constructional partial expression in the capillary loops at the site of inflammation by immunohistochemistry, suggests that endothelial activation does not involve all capillary rings at any point during the initial development of inflammation. We assume that the selectivity of the observed activation may depend on the bioavailability of specific cytokines and other mediators [32].

The expression factor Jun, also known as c-Jun, regulates catabolic transcription and apoptosis or cell death. C-Jun N-terminal kinase leads to JNK signaling cascade with Jun as its core, is activated by a range of stimuli, and is essential for cell differentiation, apoptosis, and stress response [33]. Furthermore, JNK may regulate the production of pro-inflammatory cytokines such as IL-6 to worsen PI [34]. Chemokine CXCL1 plays an essential role in the host immune response by recruiting and activating neutrophils to kill pathogens at the damaged tissue site.

Then, we combined the five genes with the online database DisGeNET and ROC validation for disease prediction. Two genes, IL-1β and HIF-1α are thought to be PI-related genes. Three genes, ICAM1, CXCL1, and JUN, were found to be potential biomarkers for PI.

The previous literature has shown that the biomarkers closely associated with the development of PI can be roughly divided into the two categories listed below. One is the plaque biofilm-associated host immune response factors, such as CD14, ClEC4E, HIF-1α, Wnt5a, etc. They regulate the activity of lipopolysaccharides, Langerhans cells, neutrophils, and macrophages, respectively, in significant ways. The second group consists of inflammatory factors (IL-1, IL-6, IL-17, TNF-α, etc.), chemokines, bone homeostasis markers (RANKL, OPG), and metalloproteinases, which are linked to supporting tissue destruction and bone resorption. They worsen the clinical symptoms of PI by a variety of mechanisms [29,35,36,37,38,39].

The current study reinforces previous findings. Similar to the results of de Araújo et al. [29] and Ishita Bhavsar et al. [40], IL-1β and HIF-1α were closely associated with PI (AUC = 0.889 and 0.667, respectively) and could be used as early markers for predicting PI [29,40]. In addition, we identified three other potential biomarkers associated with PI.

This study has several benefits. Typically, previous studies selected one or more genes of interest to compare PI to control subjects. In this study, we combined variance analyses of the entire gene expression profile, WGCNA, and Cytoscape visualization to screen the most key target genes of PI, and the results obtained are more representative. Combing whole-gene expression profiling with bioinformatics analysis may represent a future trend in PI mechanism research.

In addition, this study has some limitations. At first, the research mainly focused on data mining and data analysis, and some of the results have not been verified by experiments. We will confirm the results of this study in the future since we only obtained limited data sets and individual clinical status. We did not combine individual differences. We would achieve a more reliable result by combining more data sets and samples in a follow-up experiment.

## 5. Conclusions

In conclusion, we found five key genes from PI using WGCNA. Among them, ICAM1, CXCL1, and JUN are worthy of further study of new target genes. This provides a theoretical basis for the further exploration of the occurrence and development mechanism of PI.

## Figures and Tables

**Table 1:** GO analysis and KEGG enrichment results of DEGs (top3 according to adjusted *p* value) (Abbreviations: GO, Gene Ontology; KEGG, Kyoto Encyclopedia of Genes and Genomes; DEGs, differentially expressed genes).

Ontology	ID	Description	GeneRatio	*p* Value	P.Adjust	Qvalue	Gene Name
BP	GO:0032496	response to lipopolysaccharide	10/48	9.37 × 10^−9^	1.31 × 10^−5^	8.82 × 10^−6^	CD14/CXCL1/ICAM1/IL1B/JUN/S100A7/XBP1/ZFP36/ADAM9/LY96
BP	GO:0002237	response to molecule of bacterial origin	10/48	1.35 × 10^−8^	1.31 × 10^−5^	8.82 × 10^−6^	CD14/CXCL1/ICAM1/IL1B/JUN/S100A7/XBP1/ZFP36/ADAM9/LY96
BP	GO:0071222	cellular response to lipopolysaccharide	8/48	4.77 × 10^−8^	3.00 × 10^−5^	2.02 × 10^−5^	CD14/CXCL1/ICAM1/IL1B/XBP1/ZFP36/ADAM9/LY96
CC	GO:0031528	microvillus membrane	3/50	2.62 × 10^−5^	0.002	0.001	ITGAV/MSN/PDPN
CC	GO:0005902	microvillus	4/50	5.80 × 10^−5^	0.002	0.001	AKR1B1/ITGAV/MSN/PDPN
CC	GO:0005925	focal adhesion	7/50	6.83 × 10^−5^	0.002	0.001	ICAM1/ITGAV/MSN/S100A7/ADAM9/CAP1/TES
MF	GO:0019955	cytokine binding	5/49	2.70 × 10^−5^	0.005	0.004	IL2RG/ITGAV/ZFP36/PXDN/PDPN
MF	GO:0050839	cell adhesion molecule binding	8/49	6.16 × 10^−5^	0.006	0.005	CDH3/ICAM1/IL1B/ITGAV/MYO1B/MSN/ADAM9/TES
MF	GO:0019956	chemokine binding	3/49	9.35 × 10^−5^	0.006	0.005	ITGAV/ZFP36/PDPN
KEGG	hsa05418	Fluid shear stress and atherosclerosis	7/33	1.13 × 10^−6^	1.47 × 10^−4^	1.15 × 10^−4^	DUSP1/ICAM1/IL1B/ITGAV/JUN/NFE2L2/TXN
KEGG	hsa04064	NF-kappa B signaling pathway	5/33	5.73 × 10^−5^	0.004	0.003	CD14/CXCL1/ICAM1/IL1B/LY96
KEGG	hsa05133	Pertussis	4/33	2.41 × 10^−4^	0.010	0.008	CD14/IL1B/JUN/LY96

## Data Availability

Not applicable.
